# State of the art review of new technologies in spine deformity surgery–robotics and navigation

**DOI:** 10.1007/s43390-021-00403-6

**Published:** 2021-09-06

**Authors:** J. Alex Sielatycki, Kristen Mitchell, Eric Leung, Ronald A. Lehman

**Affiliations:** 1 Department of Orthopaedics, Center of Sports Medicine and Orthopaedics, Chattanooga, TN USA; 2 Department of Orthopaedics, Columbia University Medical Center, The Och Spine Hospital at New York-Presbyterian, New York, NY USA; 3 The Och Spine Hospital, New York-Presbyterian/The Allen Hospital, 5141 Broadway, 3 Field West, New York, NY 10032 USA

**Keywords:** Robotics, Navigation, Accuracy, Spine surgery

## Abstract

**Study design/methods:**

Review article.

**Objectives:**

The goal of this article is to review the available evidence for computerized navigation and robotics as an accuracy improvement tool for spinal deformity surgery, as well as to consider potential complications, impact on clinical outcomes, radiation exposure, and costs.

**Summary of background data/results:**

Pedicle screw and rod construct are widely utilized for posterior spinal fixation in spinal deformity correction. Freehand placement of pedicle screws has long been utilized, although there is variable potential for inaccuracy depending on surgeon skill and experience. Malpositioned pedicle screws may have significant clinical implications ranging from nerve root irritation, inadequate fixation, CSF leak, perforation of the great vessels, or spinal cord damage. Computer-based navigation and robotics systems were developed to improve pedicle screw insertion accuracy and consistency, and decrease the risk of malpositioned pedicle fixation. The available evidence suggests that computer-based navigation and robotic-assisted guidance systems for pedicle cannulation are at least equivalent, and in several reports superior, to freehand techniques in terms of accuracy. CT and robotic navigation systems do appear to decrease radiation exposure to the operative team in some reports. Published reports do indicate longer operative times with use of robotic navigation compared with traditional freehand techniques for pedicle screw placement. To date, there is no conclusive evidence that use of CT or robotic navigation has any measurable impact on patient outcomes or overall complication reduction. There are theoretical advantages with robotic and CT navigation in terms of both speed and accuracy for severe spinal deformity or complex revision cases, however, there is a need for studies to investigate this technology in these specific cases. There is no evidence to date demonstrating the cost effectiveness of CT or robotic navigation as compared with traditional pedicle cannulation techniques.

**Conclusions:**

The review of available evidence suggests that computer-based navigation and robotic-assisted guidance systems for pedicle cannulation are at least equivalent, and in several reports superior, to freehand techniques in terms of radiographic accuracy. There is no current clinical evidence that the use of navigation or robotic techniques leads to improved patient outcomes or decreased overall complications or reoperation rates, and the use of these systems may substantially increase surgical costs.

**Level of evidence:**

V.

## Introduction

Historically, spinal deformity was considered a non-surgical entity as surgeons were limited in their ability to instrument and manipulate the spinal column. Traction, casting, and other supportive care were the mainstays of care for this potentially debilitating condition. Surgical fusion for spinal deformity came about in the early 1900s with Hibbs’ use of decortication and full-body casting [1]. Over the decades, advances in spinal instrumentation techniques have allowed surgeons to gain control of the deformed spine to effect dramatic changes in alignment and body posture [1–3]. The innovation of the pedicle screw allowed for three column control, which was first described by Dr. Roy-Camille in 1963, and again later by Dr. Harrington in 1969 in a report of reducing high-grade spondylolisthesis in children [4]. The technique was still further popularized by Dr. Roy-Camille 1970, as well as at the American Academy of Orthopedic Surgeons annual meeting in 1979 [5]. The arrival of the pedicle screw significantly changed the approach to spine deformity surgery and allowed a means for substantial correction in severe deformity [6]. Furthermore, the use of pedicle screws with the constant aid of fluoroscopy evolved to freehand pedicle screw placement as our understanding of spine bony landmarks improved and numerous studies demonstrated clinical efficacy of the new technique [6–9]. Presently, pedicle screw and rod constructs have become widely utilized for posterior spinal fixation in place of the other fixation techniques (laminar hooks, sub-laminar wires, etc.) for spinal deformity correction [10].

The ability to place freehand pedicle screws allows efficiency of instrumentation with decreased radiation exposure, although mastering this art has a steep learning curve. Many studies have demonstrated reliable correlation between bony landmarks and proper pedicle screw trajectories, but the reported range of malpositioned pedicle screw using the freehand technique ranges from 1.7 to 30% [11–13]. Malpositioned pedicle screws may have significant clinical implications ranging from nerve root irritation, CSF leak, perforation of the great vessels, or even permanent spinal cord damage [12]. Computer-based navigation and robotics systems have been developed in an attempt to improve pedicle screw insertion accuracy and decreased the risk of catastrophic misses in pedicle cannulation; these technologies have continued to evolve and improve since their inception. Spine surgeons should be familiar with the various techniques for pedicle cannulation and proficient with the techniques used in surgery. The goal of this article is to review the available evidence for computerized navigation and robotics as an accuracy improvement tool for spinal deformity surgery, as well as to consider potential complications, impact on clinical outcomes, radiation exposure, and costs.

## History and utility of the navigation and robotic systems

Freehand pedicle cannulation with intra-operative fluoroscopy/plain radiographs for confirmation is a widely accepted technique, with some variation in reported accuracy. The pedicle wall breach rate of the pedicle screws using freehand technique ranges from 1.7 to 30% in the literature [13, 14]. Variations in accuracy may be explained by differences in the surgeon’s experience and technique, limitations of 2-dimensional imaging, and patient factors such as body habitus, pedicle size, and spinal deformity. Two-dimensional radiographs have limitations in assessing anatomic pedicle screw placement; thus, 3-dimensional intraoperative CT imaging may be considered to assess screw accuracy [15]. Intraoperative CT technology allows the surgeon to accurately identify misplaced pedicle screws during the procedure and replace these screws prior to the completion of the operation [16, 17]. In addition, CT-guided navigation software can be used to provide real-time 3-dimensional feedback for the surgeon. The CT navigation-based pedicle screw insertion techniques are available from several manufacturers; each system works by utilizing either a preoperative or intraoperative CT scan to digitally reconstruct an anatomic a “map” for the surgeon during the operation. This technique has gained support given the real time 3-dimenstional visual feedback obtained from the CT scan and computer reconstruction [18, 19]. Surgeons must take caution, however, against relying solely on navigational assistance, as registration inaccuracies may occur as a result of shifting of the patient or navigation array.

Robotic pedicle guidance systems were also developed around the same time as the navigations systems in the 1990s with the same goal in mind—improved accuracy/consistency in pedicle screw placement. CT navigation and robotic assistance both utilize 3-dimensional mapping of the spine to guide screw placement; the primary difference being surgeon-guided (CT navigation) vs. robotic guided execution of the pedicle screw trajectory [20].

The theoretical advantages of these new technologies are increased screw placement accuracy both in open or minimally invasive/percutaneous cases, as well as decreased radiation exposure for the operating surgeon and staff due to eliminating the use of fluoroscopy. Specifically in the setting of spinal deformity or complex revision cases with distorted anatomy, there are potential accuracy advantages in using CT or robotic navigation.

## Accuracy of CT navigation

There are numerous of studies comparing the accuracy of CT navigation to the more traditional freehand or fluoroscopically-assisted pedicle screw technique; including prospective randomized controlled trials (RCTs) and several meta-analyses comparing pedicle screw breach rates between the freehand and navigation techniques (Table 1). Xiao et al. reported a significant decrease in malpositioned pedicle screws (1.6 vs 4.2%), and all cause reoperation (5.2 vs 10.9%) in the O-arm assisted navigation system compared with freehand and/or fluoroscopic guided pedicle screw placement [21]. In 2007, Kosmopolous et al. performed a meta-analysis of over 37,000 pedicle screws, reporting overall accuracy of 95.2% for CT navigation compared to 90.3% accuracy with conventional freehand and fluoroscopy assist techniques [9]. This is one of the larger analyses available comparing the accuracy of pedicle screw placement techniques, suggesting slight superiority of CT navigation in terms of accuracy across a broad analysis. Verma et al. analyzed accuracy and complication rates of nearly 6000 pedicle screws, and reported odds ratio of 0.25 in CT navigation for neurologic complications resulting from pedicle breach (*p* = 0.07) (meaning that when pedicle breach resulted in neurologic complication, the odds that CT navigation was used in the index case was 0.25 as compared with freehand), and relative “risk” of pedicle accuracy at 1.12 for CT navigation compared to conventional techniques [22]. These results may indicate superior accuracy with CT navigation, however the observed differences are small and the overall incidence of neurologic injury from pedicle breech is low, and thus these results may not be clinically meaningful. Later in 2011, Tian et al. performed another systematic review and meta-analysis in which they reported that the odds of pedicle breach were lowest with navigated versus non-navigated techniques (odds-ratio of 0.3–0.6 for pedicle breach using CT navigation) [14]. In June of 2019, Perdomo-Pantoja et al. published a large, comprehensive meta-analysis comparing the accuracy of over 51,000 pedicle screws using the various techniques [23]. In this analysis they reported overall accuracy rates of 95.5% for CT navigation, 93.5% for freehand, 91.5% for fluoroscopy-assisted, and 90.5% for robotic assisted techniques. Interestingly, CT and robotic navigation were both superior to freehand when looking only at thoracic screw placement. These results suggest CT navigation is the most accurate, while robotic navigation was the least. Importantly, this large study included studies ranging from 1990 to 2018, thus spanning a 28-year period; thus, there is significant heterogeneity across the studies reviewed. In addition, as technology has advanced, accuracy rates with CT and robotic navigation may have changed from the earlier studies in the 1990s and early 2000s. Nevertheless, this review of over 51,000 in-vivo pedicle screws provides one of the largest to date. Specifically in cases of spine deformity, Rajasekaran et al. demonstrated a significant decrease in the pedicle cortical breach rate for the CT navigation technique at 2% compared to the traditional freehand technique at 23% breach rate in a randomized controlled trial [24]. Similarly, Laine et al. showed the pedicle breach rate to be significantly lower in the navigation group versus the freehand group (5% vs 13%) in a RCT of 100 patients randomized to either the navigation or freehand pedicle screw placement [25]. In a smaller series of pediatric deformity pedicle screws (137 screws), Luo et al. reported overall 97.8% accuracy with navigation vs. 90.9% for the freehand technique [26]. These results may indicate that navigation techniques may be especially useful when spinal anatomy is distorted in cases of deformity or revision; however there is a paucity of large studies specifically looking at navigation in cases of spinal deformity.

**Table 1:** XXX

Study	Study design	Number of screws	Outcome measures	Findings
Gao, S. Lv, Z., Fang, H. Robot-assisted and conventional freehand pedicle screw placement: a systematic review and meta-analysis of randomized controlled trials. *Eur Spine J*. April, 2017. 27(4)	Meta-analysis of randomized controlled trials (degenerative)	1360 screws: 672 FH, 688 RA	Pedicle screw accuracy	RA more accurate than FH: Relative Risk (RR) of "perfect" placement with robot 1.00–1.06. No difference in "acceptable" placement. RR of proximal facet violation 0.07 with RA. Lower radiation time with RA. Longer operative time (20 min) with RA
Fan, Y. Du, J. Liu J., et al. Accuracy of pedicle screw placement comparing robot-assisted technology and the free-hand with fluoroscopy-guided method in spine surgery: An updated meta-analysis. *Medicine.* 2018 Jun;97(22)	Meta-analysis of randomized controlled trials (degenerative)	2937 screws: 1,255 FH, 1682 RA	Pedicle screw accuracy	RA more accurate than FH: odds ratio (OR) of "perfect" placement with robot 1.38–2.07, OR of "acceptable" placement 1.17–2.08 with robotic
Kim, HJ. Jung, WI. Chang, BS. Et al. A prospective, randomized, controlled trial of robot-assisted vs freehand pedicle screw fixation in spine surgery. *Int J Med Robot.* Vol 13 (3). Sept, 2017	Randomized controlled trial (degenerative)	156 screws: 74 RA, 82 FH	Pedicle screw accuracy	No difference in accuracy between RA vs. FH. RA associated with fewer proximal facet violations
Li, HM. Zhang, RJ. Accuracy of Pedicle Screw Placement and Clinical Outcomes of Robot-Assisted Technique Versus Conventional Freehand Technique in Spine Surgery from Nine Randomized Controlled Trials: A Meta-Analysis. *Spine*. August 2019	Meta-analysis of randomized controlled trials (degenerative)	2476 screws: 1,220 RA, 1,256 FH	Pedicle screw accuracy, patient-reported outcomes	RA more accurate than FH: RR of 0.21 for grade C,D, E pedicle placement in RA vs. FH. RA with longer operative time, fewer facet violations. NO difference in patient-reported outcomes
Liu, H. Chen, W. Wang, Z. et al. Comparison of the accuracy between robot-assisted and conventional freehand pedicle screw placement: a systematic review and meta-analysis. *Int J Comput Assist Radiol Surg*. Vol 11, Iss 12. June 2016	Meta-analysis (degenerative)	1105 screws	Pedicle screw accuracy	No difference in accuracy between RA vs. FH using both open and percutaneous techniques
Marcus, HJ. Cundy, TP. Nandi, D. Robot-assisted and fluoroscopy-guided pedicle screw placement: a systematic review. *Eur Spine J*. Vol 23, Iss 2. 2014	Systematic review (degenerative)	1308 screws: 729 RA, 579 FA	Pedicle screw accuracy	No difference detected in accuracy between RA vs. FH
Park, SM. Kim, HJ. Lee, SY. Et al. Radiographic and Clinical Outcomes of Robot-Assisted Posterior Pedicle Screw Fixation: Two-Year Results from a Randomized Controlled Trial. *Yonsei Med J.* Vol 59, issue 3. 2018	Randomized controlled trial (degenerative)	78 patients	Patient-reported outcomes, adjacent segment degeneration	No difference in patient-reported outcomes or adjacent segment degeneration between RA vs. FH at 2 years postoperatively
Perdomo-Pantoja, A. Ishida, W., Zygourakis, C. Et al. Accuracy of Current Techniques for Placement of Pedicle Screws in the Spine: A Comprehensive Systematic Review and Meta-Analysis of 51,161 Screws. *World Neurosurg*. Vol 126, Jun 2019	Systematic review and meta-analysis (degenerative)	51,161 screws	Pedicle screw accuracy	Accuracy rates were 95.5%, 93.1%, 91.5%, and 90.5%, via CTNav, FH, FA, and RA techniques, respectively. RA and CTNav were associated with the highest PS accuracy in the thoracic spine, compared with FH
Rajasekaran, S. Vidyadhara, S. Ramesh, P. Shetty, AP. Randomized clinical study to compare the accuracy of navigated and non-navigated thoracic pedicle screws in deformity correction surgeries. *Spine*. Vol 32, Issue 2. Jan 2007	Randomized controlled trial (deformity)	478 thoracic screws: 242 CTNav, 236 FA	Pedicle screw accuracy	CTNav more accurate (2% breach rate) than flouroscopy-assist (23% breach rate) in thoracic deformity cases
Kosmopolous, V. Schizas, C. Pedicle screw placement accuracy: A meta-analysis. *Spine*, Vol 32 (3), Feb 2007	Meta-analysis (degenerative)	37,337 screws	Pedicle screw accuracy	CT navigation accuracy 95.2%, "Conventional" screw accuracy 90.3%
Staartjes, VE. Klukowska, AM. Schroder, ML. Pedicle Screw Revision in Robot-Guided, Navigated, and Freehand Thoracolumbar Instrumentation: A Systematic Review and Meta-Analysis. *World Neurosurg.* May 2018	Meta-analysis (degenerative)	7095 patients	Incidence of pedicle screw revision	No difference in Intra-operative screw revisions in RA, CTNav, and FH. Postoperative revisions lower in RA (OR, 0.3; 95% CI, 0.1–0.9;* P* = 0.04) and CTNav (OR, 0.3; 95% CI, 0.2–0.5;* P* < 0.001)
Tian, NF. Huang, QS. Zouh, P. Et al. Pedicle screw insertion accuracy with different assisted methods: a systematic review and meta-analysis of comparative studies. *Eur Spine J*. Vol 20, iss 6. June 2011	Systematic review and meta-analysis (degenerative)		Pedicle screw accuracy	CTNav more accurate (OR 0.3–0.6 for pedicle violation vs. non-navigated technique)
Yu, L. Chen, X. Margalit, A. Et al. Robot-assisted vs freehand pedicle screw fixation in spine surgery—a systematic review and a meta-analysis of comparative studies. *Int J Med Robot*. Feb 2018	Systematic review and meta-analysis (degenerative)	3625 screws	Pedicle screw accuracy	No significant difference in accuracy between RA and FH (95.5% compared with 92.9%;* P* = 0.51). No difference in complication rate. RA associated with longer O.R. time
Verma, R. Krishan, S. Haendimayer, K. Mohsen, A. Functional outcome of computer-assisted spinal pedicle screw placement: a systematic review and meta-analysis of 23 studies including 5,992 pedicle screws. *Eur Spine J*. Vol 19, Iss 3. March 2010	Systematic review and meta-analysis (degenerative)	5992 screws	Pedicle screw accuracy, neurologic complications	Neurologic complications OR 0.25 (95% CI 0.06, 1.14) in favor of CTNav (p = 0.07). CTNav more accurate than conventional pedicle screw insertion with a relative risk of 1.12 (95% CI 1.09, 1.15) (*p* < 0.00001)
Gelalis, ID. Paschos, NK. Pakos, EE. Et al. Accuracy of pedicle screw placement: a systematic review of prospective in vivo studies comparing free hand, fluoroscopy guidance and navigation techniques. *Eur Spine J.* Vol 21, Iss 2. Feb 2012	Systematic review (degenerative)	6617 screws	Pedicle screw accuracy	FH completely within pedicle in 69 to 94%, FA 28 to 85%, CTNav 89 to 100%

The available evidence is insufficient to make the claim that CT navigation is definitively superior to traditional freehand technique in all surgeons’ hands. There is a wide variation in reported freehand pedicle screw accuracy, which is likely explained by variation in surgeon training, experience, and case complexity. To be clear, the studies analyzing the accuracy of pedicle cannulation are not able to fully account for variations in surgeon skill and knowledge of applied anatomy. Certainly a skilled and experienced surgeon may demonstrate equal or even better accuracy with the freehand over the CT navigation technique. Where CT navigation may have potential to outperform freehand techniques is in the setting of severely distorted anatomy, revision surgery, or for surgeons with less experience and training with traditional techniques.

## Accuracy of robotic assistance

Robotic-assisted pedicle cannulation techniques have become more widely available in recent years; and although the majority of spine surgeons do not have this technology available, the use of robotics is growing. Consequently, there is a rapidly-growing number of studies assessing this new technology in regards to accuracy, efficiency, and outcomes (Table 1). A retrospective study in 2011 by Kantelhardt et al. found that 94.5% of robotic-guided screws were accurately placed relative to 91.4% in the freehand group, a difference that was statistically significant [27]. This study also showed that there was no difference in pedicle screw accuracy for the robotic assisted screws in both the open and percutaneous pedicle screw placements, which implies similar precision of the robot regardless of which surgical approach was used (open versus percutaneous). Schatlo et al. reported 83.6% “perfect” pedicle screw placement with robotic assistance compared to 79% with the freehand technique using the Gertzbein–Robbins classification for pedicle screw accuracy [28]. In contrast to the findings in these smaller studies that robotic navigation is more accurate, Kim et al. performed a randomized controlled trial of robot-assisted versus freehand pedicle screw placement and found no difference in pedicle cannulation accuracy and fewer proximal facet violations with robotic assistance [29].

Several large meta-analyses reviewing the accuracy of robotic assistance have also been published in recent years. Gao et al. reported a small improvement in “perfect” pedicle screw placement (relative “risk” 1.00–1.06) for robotic versus freehand cannulation in their meta-analysis. [30] In a large meta-analysis of level 1 randomized-controlled trials, Fan et al. reported on the results of 2,937 screws (1255 freehand and 1682 robotic-assisted) [31]. In this report the odds ratio for “perfect” pedicle screw placement was 1.38–2.07 for robotic assistance, and the odds of “acceptable” pedicle placement (less than 1 mm cortical breach) was 1.17–2.08 for robotic assistance. In agreement with these findings, Li et al. reported robotic assistance was more accurate than freehand with a relative risk of 0.21 for grade C, D, or E pedicle placement in robotic assistance [32].

In contrast to these analyses reporting increased accuracy with robotic assistance compared with the freehand technique, there are several reports that did not identify any significant differences in the two methods. In a systematic review and meta-analysis of 1105 pedicle screws, Liu et al. found no difference in accuracy between the freehand and robotic techniques [33]. Similarly, Marcus et al. found no difference in accuracy in their analysis of 1308 pedicle screws placed with robotic versus freehand techinique [34]. Finally, in a large analysis of 3,625 pedicle screws, Yu et al. reported no difference in accuracy with robotic assistance (95.5% versus 92.9% in the freehand technique, *p* = 0.5) and longer operative time with robotic assistance [35].

In general, meta-analyses such as those reviewed here should be considered only as strong as the studies they review. Indeed, there is variability across study methods and results analysis that may confound the findings of studies attempting to pool results together. However, the simplistic nature of the question of pedicle screw accuracy (i.e. is the screw contained within the pedicle or not on CT imaging, and is there a cortical breach?) does lend itself to larger pooled analysis. Thus, meta-analyses of pedicle screw accuracy are a reasonable means of assessing a large number of screws across multiple studies. There is also value in assessing accuracy across difference surgeons and centers to address the question of reproducibility of accuracy using robotic systems. Thus, there is compelling evidence in the literature that CT navigation and robotic assistance are at least equivalent, and in many reports superior, to freehand techniques in terms of accuracy of pedicle screw placement. One significant criticism is that the studies demonstrating the accuracy of robotic systems are relatively small and in most cases retrospective given the early stages of these technologies. Several of the meta-analyses cited in this review do help to combat this critique. However, at this time there is insufficient evidence to prove that robotic pedicle cannulation is more accurate than traditional techniques in all surgeons’ hands. The available evidence does seem to corroborate that robotic systems are at least as accurate as tradition techniques and are thus safe for clinical use with surgeons who are properly trained and experienced with this technology.

## Clinical outcomes

Comparing the accuracy of the various pedicle cannulation techniques is a relatively straightforward endeavor. Demonstrating improvement in clinical outcomes related to CT navigation or robotic assistance is a much more significant challenge. Some studies in degenerative cases have shown decreased re-operation rates specifically for the problem of screw malposition with the use of navigation technology, although there are no studies reporting decreased re-operation rates for all causes. Aside from this there is no evidence in the literature that shows a demonstrable change in long-term patient-reported or clinical outcomes with the use of CT navigation or robotic assistance as compared with traditional / freehand techniques for pedicle screw insertion. To our knowledge no studies have determined clinical outcome differences in primarily deformity versus degenerative cases with the use of navigation / robotics.

Xiao et al. reported a significant decrease in malpositioned pedicle screws (1.6 vs 4.2%), and all cause reoperation (5.2 vs 10.9%) in the O-arm-assisted navigation system compared with freehand and/or fluoroscopic-guided pedicle screw placement [21]. The same group also demonstrated decreased hospital stay (4.72 vs 5.43 days), and readmission rate (0.8 vs 4.2%) for the navigation system group; however, it is likely that this difference was related to surgical approach (MIS vs. open) and not necessarily due to the use of navigation in itself. Staartjes, et al. performed a study analyzing pedicle screw revision rates across the different techniques. In this report they found no difference in intra-operative screw revisions between CT navigation, robotic assistance, and freehand technique. They did, however, identify a lower rate of postoperative revisions for screw malposition in the robotic and CT navigation cohorts (odds ratio 0.3 compared with freehand technique—indicating that in the specific cases of revision for screw malposition, the odds of use of navigated techniques in the original surgery was 0.3 versus the odds that freehand was used). In sensitivity analysis, statistical significance for this finding was lost for the robotic cohort but not for the CT navigation cohort. Importantly, the overall rate of reoperation for screw revision was relatively low at 2.1% of those cases requiring reoperation; thus, while there may be a statistical improvement in screw malposition, revision rates the number needed to treat is likely to be very high. This calls into question whether the high acquisition and maintenance costs associated with CT navigation and robotic technology warrant a clinically small reduction in re-operations for screw malposition.

One of the other purported benefits of robotic and CT navigation is the potential for decreased proximal facet joint violation in lumbar fusion cases. Indeed, several of the previously-cited studies have shown a decreased rate of proximal facet violation with the use of navigated or robotic techniques [29, 30, 36, 37]. Importantly, it should be noted that proximal facet joint violation may be mitigated with the use of CT or robotic navigation in the setting of MIS / percutaneous instrumented cases; when open techniques are used, the proximal facet may be directly visualized and thus avoided. Despite the findings indicating improved pedicle screw placement, large meta-analyses have shown a lack of statistically significant differences in clinical outcomes [22].

CT navigation and robotic assistance have shown improved pedicle screw placement accuracy compared to the freehand technique in numerous studies. Indeed, Verma et al. did report a lower rate of pedicle screw-related neurologic complications using CT navigation (odds ratio 0.25); which would suggest the potential for at least small potential improvement in patient outcomes [22]. To contrast, the report by Yu et al. did not show any difference in complication rates for robotic assistance versus traditional techniques; although robotic navigation was associated with longer operative times [38]. In 2018, Park et al. reported on the 2-year radiographic and clinical results of a randomized controlled trial for robotic versus traditional pedicle screw cannulation [39]. In this report of 78 patients, there was no measurable difference in patient-reported outcomes or rates of adjacent segment degeneration or disease among the patient cohorts. It should therefore be stated that true clinical superiority for CT or robotic navigation has not been demonstrated [35, 40].

## Navigation in spinal deformity

The available evidence is insufficient to make the claim that CT navigation is definitively superior to traditional freehand technique in all surgeons’ hands. In addition, there are no studies demonstrating the superiority of navigated techniques specifically in the setting of spinal deformity, although anecdotally the use of navigated techniques may be a valuable aid to the surgeon in complex spine cases. Overall, there is a wide variation in reported freehand pedicle screw accuracy, which is likely explained by variation in surgeon training, experience, and case complexity. Certainly, a skilled and experienced surgeon may demonstrate equal accuracy with the freehand or CT navigation technique. CT navigation likely has the most potential to outperform freehand techniques is in the setting of severely distorted anatomy, complex deformity surgery, revision deformity surgery, and with pelvic fixation. A thorough understanding and appreciation of the patient’s spine in complex deformity cases is essential for the spine surgeon, but the 3D nature of the deformed spine is often difficult to visualize for even the most experienced deformity surgeon. Many studies have shown high pedicle breach rate in spinal deformity cases using the freehand technique, which implies the unreliable appreciation of the complex spine without additional technology to supplement the surgeon’s understanding. Gelais et al. reviewed more than 25 prospective studies comparing the freehand technique, 2D fluoroscopy-assisted navigation system and CT-based navigation system and showed the accuracy of the CT-based navigation group to be 89–100% [18]. In this review, there was high variability of the freehand technique (69–94% accuracy) and 2D fluoroscopy-assisted navigation system (28–85%), which may be secondary to the performing surgeon’s experience and the difficulty of the case. Although the freehand technique may achieve low breach rate in certain studies, the consistency and the accuracy of pedicle screw placement using the CT-based navigation system is noteworthy. Other meta-analysis have further corroborated these findings, which demonstrated lower pedicle screw malposition rates in 3D-fluroscopic navigation systems compared to the traditional freehand technique [10, 20, 27].

The advantage of CT-based navigation system is potentially even more pronounced in pediatric deformity cases which often have severe thoracolumbar distortion, frequently with smaller pedicles. Luo et al. reported an impressive 97.8% pedicle screw accuracy rate for such population in 137 screws for 16 pediatric spinal fusions, which is an improvement from 90.9% accuracy rate of freehand techniques [28, 29].

Robotic-assisted pedicle cannulation techniques became available several years after the navigation-based systems, and there are a growing number of studies assessing this new technology in regards efficacy, safety and efficiency. A retrospective study in 2011 by Kantelhardt et al. found that 94.5% of robotic-guided screws were accurately placed relative to 91.4% in the freehand group, a difference that was statistically significant [30]. This study also showed that there was no difference in pedicle screw accuracy for the robotic-assisted screws in both the open and percutaneous pedicle screw placements, which implies similar precision of the robot regardless of which surgical approach was used (open versus percutaneous). To further support the previous study, Schatlo et al. reported 83.6% “perfect” pedicle screw placement with robotic assistance compared to 79% with the freehand technique using the Gertzbein–Robbins classification [31]. It should be noted that these studies reviewed all cases of robotic instrumentation and did not delineate between degenerative and deformity procedures.

## Pelvic fixation

For many spine surgeons, an in-depth and 3-demensional understanding of complex pelvic anatomy may not be as complete as their grasp of spinal anatomy. It thus stands to reason that navigational and/or robotic techniques may be a useful adjunct in instrumenting the pelvis. Additional studies have demonstrated the accuracy of robotic assisted S2-Alar-Illiac screw placements ranging from 85% to nearly 100% [32, 33]. Contrary to some of these initial reports, some meta-analysis suggest insufficient evidence to promote screw placement superiority of robotic systems compared to both the freehand and navigation system [34, 35]. At the author’s institution we have recorded 94% accuracy with S2-Alar-Iliac cannulation using robotic assistance. Our experience has been that robotic assistance is particularly useful in the setting of instrumenting a previously-operated spine when the dorsal anatomy is severely distorted. Particularly in revision deformity operations, there is often a thick sheet of fusion mass bone that distorts the dorsal anatomy and hinders the surgeon from using standard freehand techniques for pedicle screw insertion. In these cases, we have found that use of the robot has actually shown slightly decreased operative time per vertebral level (66 min vs. 55 min in primary vs. revision cases, respectively) when compared to primary cases (unpublished data) (comparison of robot vs. freehand in revision cases). Essentially, with the preoperative CT and planning software the surgeon can map out the trajectory of each pedicle screw, then during the case the robotic arm guides the start point and thus makes screw insertion quick and efficient, without the need for repeat fluoroscopic images to confirm accurate placement.

There is compelling evidence in the literature that CT navigation and robotic assistance are at least equivalent, and in many reports superior, to freehand techniques in terms of accuracy. Most of the studies demonstrating the efficacy of robotic systems are relatively small and are retrospective given the early stages of these technologies. A definitive answer to the advantage of using these advanced techniques compared to the freehand screw insertion will require large prospective studies in the future.

## Practical benefits of CT navigation and robotics

CT navigation and robotic assistance may provide significant advantages for spinal deformity surgeons, although there is a paucity of data available on the accuracy of robotic navigation specifically in the setting of spinal deformity or in revision fusion. In an accuracy analysis of S2-Alar-Iliac screw placement, Shillingford et al. did report similar accuracy rates between freehand (94.9%) and robotic (97.8%) techniques [41]. Similarly, Laratta et al. also reported on 46 robotic-assisted S2 Alar-Iliac screws placed in vivo, with only 2 breaches as seen on CT that did not cause any negative effects, thus demonstrating that robotic navigation may be a useful tool for spino-pelvic fixation in deformity operations [42]. Anecdotally, the authors have found robotic and/or CT navigation to be particularly helpful in instrumenting spines with distorted anatomy from deformity or prior fusion. The ability to see the 3-dimensional anatomy of the spine in real time can certainly be a useful tool in such scenarios. However, there is no available data proving that navigational assistance improves screw accuracy in extreme deformity or revision settings.

Finally, robotic assistance and CT navigation have also been shown to reduce the incidence of proximal facet joint violation in several of the large studies cited previously. Gao et al. reported relative risk of 0.7 for proximal facet violation using robotic navigation [30]. Similar findings were corroborated by Li et al. [32]. Despite these findings, no studies to date have been able to demonstrate any reduction in adjacent segment disease with the use of navigation or robotic techniques; likely because proximal facet violation is only one of numerous factors that may contribute to adjacent segment disease in spinal fusion.

## Radiation considerations

Radiation exposure to the surgeon and O.R. team during spine surgery is another primary criticism of freehand technique that often requires multiple fluoroscopic images for confirmation. The three dimensional navigation systems have been shown to significantly decreased the radiation exposure for the surgeon compared to the freehand technique [27, 30, 43]. Roser et al. demonstrated that radiation time for the robotic system was 16.0 s compared to the 31.5 s for the traditional freehand technique [44]. In addition, CT navigation and robotics have been demonstrated to be safely used with the patient in the lateral decubitus position; which can thus significantly reduce operative time in cases of anterior interbody fusion with posterior screw augmentation [45–47]. In light of these findings, we may conclude that CT and robotic navigation may decrease radiation exposure for the surgeon, however radiation exposure may not be decreased (and may even be increased) for the patient due to the use of intraoperative or preoperative CT scan. Mendelsohn et al. have in fact reported that the use of CT navigation increases radiation exposure to the patient, while at the same time decreasing radiation exposure to the surgeon and operative team [48]. Certainly decreased radiation exposure for the surgical team is a significant benefit, however this comes at the expense of increased radiation to the patient and thus the surgeon should be cognizant of patients with unique increased risks to radiation exposure (children, for example).

## Limitations of navigation and robotics

The most substantial purported benefits of CT navigation and robotics are increased pedicle screw accuracy/consistency, reduced radiation exposure to O.R. staff (but not the patient), and potential for improved accuracy in deformity and revision spinal fusion. Despite these potential advantages there are substantial limitations to consider with use of this technology. Foremost among these limitations is the consideration for cost. One of the available robotic systems is generally sold for around $1 million U.S. dollars, a significant expense for any hospital system. Particularly for those surgeons with experience and proficiency using freehand techniques, the addition of CT navigation or robotic technology may increase O.R. time and cost without imparting a meaningful clinical benefit in terms of improved patient outcomes [49]. Indeed, several of the large studies cited in this review have reported increases in operative time and cost associated with the use of robotics [30, 32] Over time it may become evident that increased accuracy with these advanced instrumentation tools may improve complication and re-operation rates related to malpositioned screws as well as reduce total operative time, particularly with distorted spine anatomy. However, to date there are no studies that demonstrate superior cost-effectiveness of CT navigation and robotics in spine surgery. Further still, many of the studies published to date which promote the benefits of CT navigation (big industry) and/or robotic technologies (smaller start-ups) are industry-driven and authored by surgeons with potential conflicts of interest that should be considered in interpreting their results.

In addition, complete reliance on CT navigation or robotics may result in the surgeon being beholden to the technology itself. As with any computer or mechanical system, there is always the potential for system malfunction or failure. Over-reliance on navigation technologies, especially during surgical training, may compromise the applied anatomy skills of future generations of surgeons. If the treating surgeon is not familiar with more traditional instrumentation techniques, malfunction of these assistive technologies may result in the need for canceling or aborting surgery; which is of course not a favorable circumstance for the patient or surgical team. If the treating surgeon is familiar with the traditional freehand technique, a malfunction of CT navigation or robotics need not lead to a major alteration in the patient’s care. Ideally, the surgeon should regard CT navigation and robotic assistance as a tool for use in instrumenting the spine, but should not rely on this technology to replace surgical experience, a detailed understanding of spine anatomy, and sound clinical judgment. While the potential benefits of navigation are real, the potential loss of surgical skill and applied anatomy understanding is a true detriment that must be guarded against.

## Conclusions

**Fig. 1:**
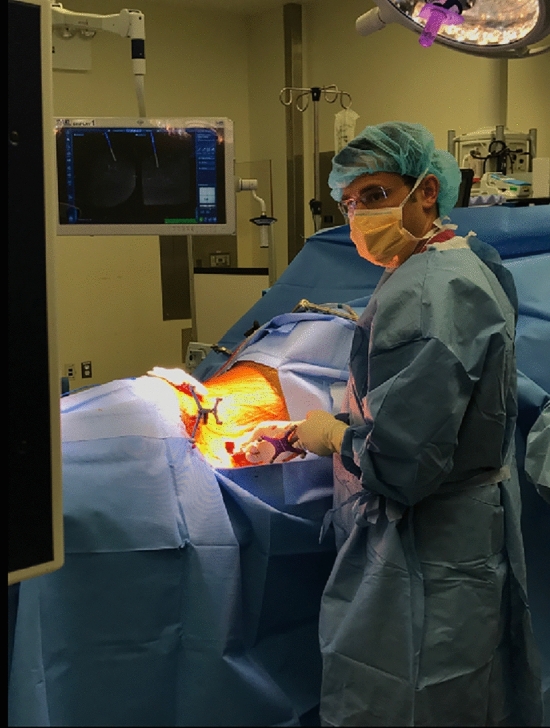
Percutaneous pedicle screw placement with the patient in lateral decubitus position performed using the CT based 3-dimensional respresentation of the patient’s spine in the navigations system

**Fig. 2:**
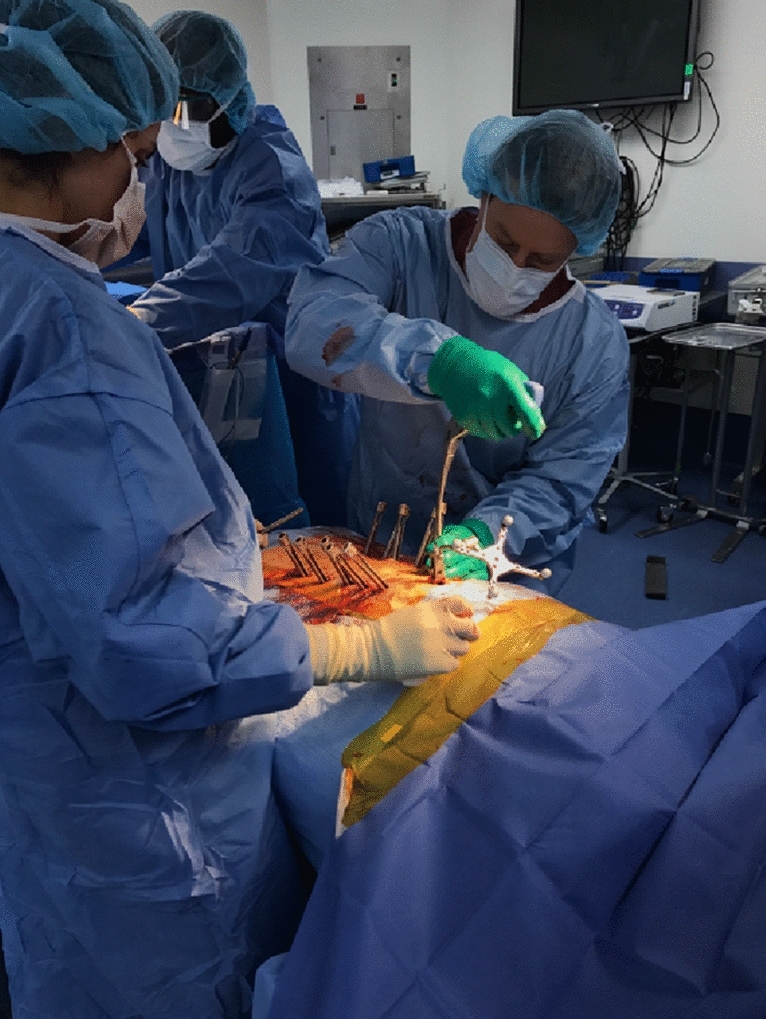
Multilevel percutaneous pedicle cannulation performed using the navigations system. This technique allows reduction in blood loss and paraspinal muscle damage

**Fig. 3:**
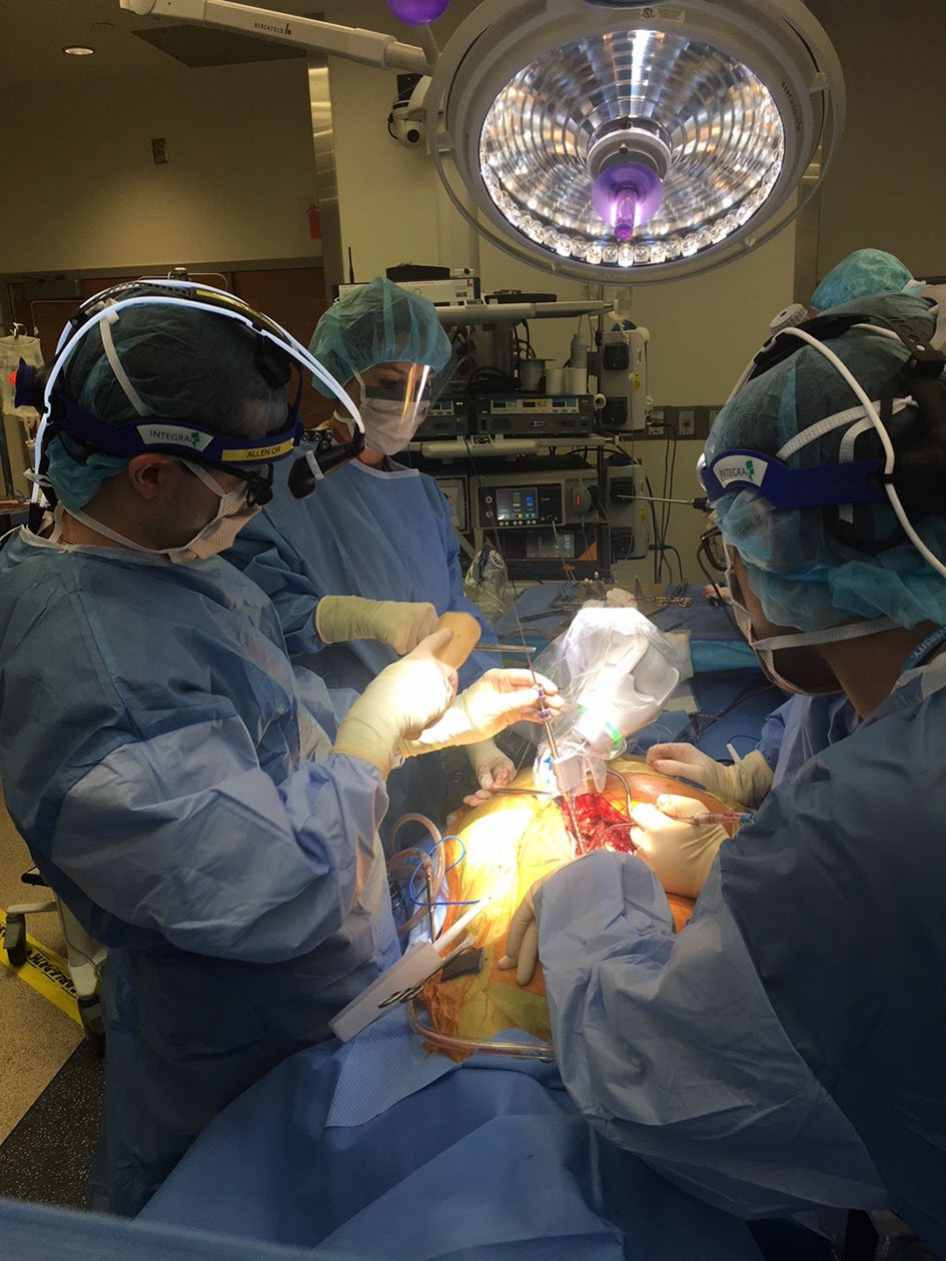
Guidewire insertion using Robotics system for preplanned pedicle screw insertion

**Fig. 4:**
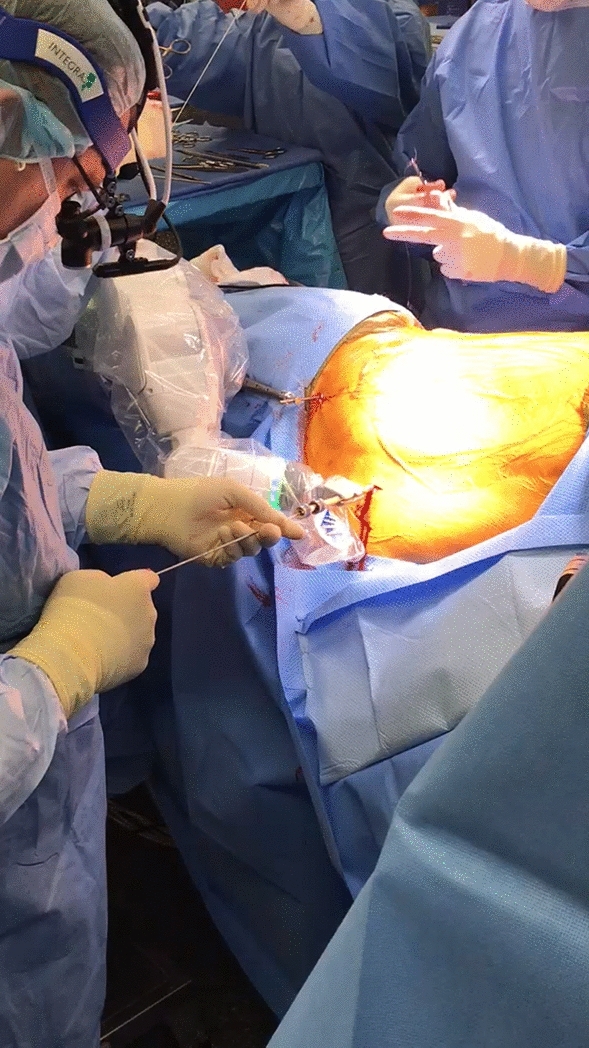
Percutaneous guidewire insertion for preplanned pedicle screw insertion in the lateral decubitus position

The available evidence suggests that computer-based navigation and robotic-assisted guidance systems for pedicle cannulation are at least equivalent, and in several reports superior, to freehand techniques in terms of accuracy. CT and robotic navigation systems do appear to decrease radiation exposure to the operative team; although most reports do indicate longer operative times with use of robotic navigation compared with traditional/freehand techniques for pedicle screw placement. There are theoretical advantages with robotic and CT navigation in terms of both speed and accuracy for severe spinal deformity or complex revision cases, however there is a need for studies investigating this technology in these specific cases, as the use of navigation has not been robustly studied in these scenarios. On the negative side, CT navigation and robotics are associated with significant costs, and thus far have not been demonstrably cost effective. Clinical superiority with use of CT or robotic navigation has not been shown in any of the studies to date, and thus their use must be carefully considered in light of the potential costs. Where the studies have shown the potential for improved radiographic accuracy, the question of whether this meaningfully solves a true clinical problem is not yet answered. In addition, excessive reliance on navigational assistance has the potential to compromise the applied anatomy skills of surgeons both in training and in practice. System malfunctions and technology failures may significantly alter a patient’s care or even lead to harm if navigation malfunction is not recognized or if the treating surgeon is unable to complete the operation with standard techniques. Finally, to date there is no conclusive evidence that use of CT or robotic navigation has any measurable impact on patient outcomes or reduction in overall complication rates (Figs 1, 2, 3, 4).
